# Diagnostic Method Advancement to Improve the Prognosis of Pancreatic Ductal Adenocarcinoma

**DOI:** 10.3390/diagnostics16142286

**Published:** 2026-07-22

**Authors:** Pradez Sapkota, Masataka Kikuyama, Goro Honda

**Affiliations:** 1 Department of Hepatobiliary-Pancreatic Surgery and Liver Transplantation, Nepal Mediciti Hospital, Nakkhu, Lalitpur 44600, Nepal; spradez@gmail.com; 2 Department of Gastroenterology, Tokyo Women’s Medical University, 8-1, Kawada-cho, Shinjuku-ku, Tokyo 162-8666, Japan; 3 Department of Surgery, Tokyo Women’s Medical University, 8-1, Kawada-cho, Shinjuku-ku, Tokyo 162-8666, Japan; honda.goro@twmu.ac.jp

**Keywords:** focal pancreatic parenchymal atrophy, pancreatic cancer, carcinoma in situ, high grade pancreatic epithelial neoplasia, serial pancreatic juice aspiration cytologic examination

## Abstract

Pancreatic ductal adenocarcinoma (PDAC) has a poor prognosis, and early diagnosis is essential for improving patient outcomes. Conventional imaging modalities such as ultrasonography, computed tomography (CT), and magnetic resonance imaging (MRI) have limitations in detecting small PDAC. Endoscopic ultrasonography (EUS) is useful for identifying small lesions; however, even small PDAC can be invasive and may metastasize, resulting in Stage IV disease. The most effective way to improve the prognosis of PDAC is to diagnose lesions confined to the ductal epithelium, such as high-grade pancreatic intraepithelial neoplasia (HG-PanIN) or carcinoma in situ (CIS). Identifying focal pancreatic parenchymal atrophy (FPPA) on CT or MRI may suggest the presence of HG-PanIN/CIS and prompt further evaluation using serial pancreatic juice aspiration cytologic examination (SPACE). Recognizing FPPA and performing SPACE enables the diagnosis of PDAC at Stage 0 (HG-PanIN/CIS), which can lead to improved prognosis.

## 1. Introduction

Pancreatic cancer is one of the leading causes of cancer-related deaths worldwide [1]. Klein reported that smoking remains a major risk factor for pancreatic cancer worldwide, while the increasing prevalence of diabetes and obesity is expected to further increase its incidence [1]. In addition, accumulating evidence suggests that heavy alcohol consumption contributes to pancreatic cancer risk. Furthermore, growing knowledge of inherited genetic factors indicates that they may account for approximately 22–33% of the overall risk of pancreatic cancer.

The overall five-year survival rate for pancreatic cancer is approximately 10% worldwide, representing only a modest improvement over the past decade [2]. According to Leiphrakpam et al. [2], this poor survival is primarily attributable to late-stage diagnosis and the lack of effective screening methods. At present, population-based screening for asymptomatic individuals is not recommended, emphasizing the importance of identifying and closely monitoring individuals at high risk for pancreatic cancer.

Illic et al. reported that pancreatic cancer remains one of the deadliest malignancies [3]. The mortality-to-incidence (M/I) ratio is approximately 98% [4]. Furthermore, survival rates differ only slightly between developed and developing countries.

According to the National Cancer Center, Japan, the five-year survival rates for male and female patients diagnosed with pancreatic cancer between 2012 and 2015 were 10.7% and 10.2%, respectively [5]. These rates are among the lowest of all cancer types, excluding childhood cancers.

However, the actual long-term prognosis has been reported to be less than 5% [6]. Estimates of short-term survival are generally reliable because of the high rate of early disease-related mortality, whereas estimates of long-term survival remain limited. Moreover, survival estimates may be overestimated when there is substantial loss to follow-up. Differences in patient characteristics between clinical trials and real-world registries also influence survival outcomes, with patients enrolled in clinical trials demonstrating markedly better survival than those included in national registry databases [6].

Several anatomical, biological, and clinical factors contribute to the poor prognosis of pancreatic cancer. Major blood vessels, including the celiac artery, superior mesenteric artery, and portal vein, are located in close proximity to the pancreas. Pancreatic ductal adenocarcinoma (PDAC) readily invades these vessels, frequently rendering the tumor unresectable [7]. The PDAC tumor microenvironment consists of pancreatic stellate cells (PSCs), fibroblasts, inflammatory cells, endothelial cells, nerve cells, and other stromal components [8,9]. PSCs are the predominant cellular component of the tumor stroma. Although they remain quiescent in normal pancreatic tissue, they become activated by cytokines and growth factors, including tumor necrosis factor-α (TNF-α), transforming growth factor-β (TGF-β), and interleukins (IL-1, IL-2, and IL-10) [8]. Activated PSCs produce abundant extracellular matrix (ECM) proteins, such as collagen, fibronectin, and laminin, resulting in the formation of dense fibrotic stroma. This desmoplastic tumor microenvironment plays a crucial role in the development of chemoresistance in pancreatic cancer [10,11], although combination chemotherapy regimens such as folinic acid/fluorouracil/irinotecan/oxaliplatin (FOLFIRINOX) and gemcitabine plus nab-paclitaxel have significantly improved the prognosis of patients with advanced pancreatic cancer [10,11].

Early diagnosis of pancreatic cancer remains challenging using conventional imaging modalities, including ultrasonography (US), computed tomography (CT), 18F-fluorodeoxyglucose positron emission tomography/computed tomography (FDG-PET/CT), and magnetic resonance imaging (MRI) [1,2,12].

## 2. Conventional Diagnostic Methods

### 2.1. Ultrasonography

Ultrasonography (US) is a noninvasive, inexpensive, and widely available imaging modality commonly used as the initial examination for patients with abdominal symptoms and as part of the routine evaluation of abdominal diseases [13]. It is also frequently used during medical checkups for asymptomatic individuals.

Yamaguchi et al. reported that patients with pancreatic ductal adenocarcinoma (PDAC) detected by US during routine medical checkups had a significantly better prognosis than those diagnosed after the onset of symptoms, suggesting that US screening of asymptomatic individuals may contribute to earlier diagnosis and improved clinical outcomes [14].

However, the diagnostic performance of US for PDAC is limited because the pancreas is located deep within the abdomen, posterior to the stomach, making visualization difficult. To overcome this limitation, several techniques have been proposed. Ashida et al. demonstrated that filling the stomach by having patients drink milk tea improved the visualization of pancreatic tail tumors [15]. Likewise, Sofuni et al. recommended changing the patient’s body position or having patients drink degassed water to improve pancreatic visualization [16]. In the right lateral decubitus position, gastric gas shifts toward the fundus while the pancreas moves toward the midline, facilitating visualization of the pancreatic tail. Similarly, the semi-sitting position improves visualization by altering the position of the abdominal organs.

Despite these technical improvements, the diagnostic sensitivity of US remains limited, particularly for small tumors. A multicenter study conducted in Japan reported a sensitivity of only 67.3% for PDAC measuring 20 mm or less [17]. Nevertheless, US remains valuable because it can detect indirect findings suggestive of PDAC, including main pancreatic duct dilatation and pancreatic cystic lesions [16], which may prompt further diagnostic evaluation.

### 2.2. Computed Tomography

Computed tomography (CT) is one of the most widely used imaging modalities for evaluating abdominal diseases. In particular, contrast-enhanced CT (CECT) plays a central role in the diagnosis of both acute abdominal disorders and malignant tumors [18]. CECT provides valuable information for identifying the causes of acute abdominal pain, including intestinal perforation, appendicitis, peritonitis, hepatitis, cholecystitis, bowel obstruction, hernia, and vascular occlusion, while also allowing for a comprehensive assessment of abdominal organs for malignant lesions [18].

CT demonstrates high diagnostic accuracy and sensitivity for pancreatic tumors larger than 2 cm [19]. However, its sensitivity decreases substantially for tumors smaller than 2 cm, ranging from 69% to 78% [19,20,21,22].

Kurita et al. reported that dual-energy contrast-enhanced CT (DECT) improved the visualization of small pancreatic cancers, with delayed-phase 50-keV images providing superior tumor-to-parenchyma contrast [23]. These findings suggest that DECT may facilitate the detection of early-stage pancreatic cancer. However, the median tumor size in that study was 22 mm (range, 10–70 mm), indicating that further evaluation in smaller lesions is warranted.

LeBlanc et al. demonstrated that a tumor size ≤2 cm is a major factor limiting the sensitivity of CECT for detecting PDAC [24]. They concluded that improving the conspicuity of small pancreatic tumors on CECT remains an important challenge.

Some PDACs are not directly visible on cross-sectional imaging because of their small size or attenuation, similar to that of the surrounding pancreatic parenchyma. In such cases, secondary imaging findings—including upstream main pancreatic duct dilatation caused by abrupt ductal stenosis, the double-duct sign, focal pancreatic atrophy, and subtle delayed enhancement—are important diagnostic clues that should prompt further investigation [21,25,26].

### 2.3. 18F-Fluorodeoxyglucose Positron Emission Tomography/Computed Tomography

Numerous studies have evaluated the diagnostic performance of 18F-fluorodeoxyglucose positron emission tomography/computed tomography (FDG-PET/CT) in pancreatic cancer [27,28]. Overall, FDG-PET/CT has demonstrated high sensitivity and specificity for detecting PDAC. However, most studies have included tumors larger than 2 cm [27,28], which are generally detectable by conventional imaging modalities.

FDG uptake is closely associated with tumor size. In particular, the degree of 18F-FDG accumulation increases with increasing lesion size, whereas the accurate evaluation of lesions smaller than 1 cm is hampered by the partial-volume effect, making the measurement of standardized uptake values (SUVs) unreliable [27]. Consequently, the detection of very small pancreatic cancers using FDG-PET/CT remains challenging.

Although FDG-PET/CT has limitations in the detection of early-stage disease, it provides excellent diagnostic performance for identifying distant metastases and therefore plays an important role in the staging of PDAC [29,30].

In addition, several FDG-PET/CT-derived quantitative parameters have been investigated as prognostic biomarkers in PDAC [31]. However, Pu et al. concluded that no single PET parameter has yet been established as the optimal prognostic indicator, and large multicenter prospective studies are required to validate their clinical utility.

### 2.4. Magnetic Resonance Imaging

Magnetic resonance imaging (MRI) is another important imaging modality for the diagnosis of pancreatic ductal adenocarcinoma (PDAC). MRI has a diagnostic sensitivity and specificity comparable to those of multidetector computed tomography (MDCT) for the staging of PDAC [25]. However, it has not been shown to be superior to contrast-enhanced CT (CECT) for the primary detection of PDAC [32,33]. Rather, MRI is particularly valuable as a problem-solving tool for pancreatic lesions that are isoattenuating on MDCT [25].

Kim et al. reported that visually isoattenuating pancreatic adenocarcinomas on CT were detected by MRI with a sensitivity of 79.2% [26]. In their study, the maximum diameter of MRI-visible lesions ranged from 1.2 to 3.7 cm (median, 2.5 cm). They concluded that MRI has moderate sensitivity for detecting visually isoattenuating pancreatic adenocarcinomas and is useful as a complementary imaging modality when PDAC is suspected despite inconclusive CT findings [26].

Similarly, Kanno et al. reported detection rates for Stage I PDAC (<2 cm) of 65.8% with CT and 57.5% with MRI [17]. These findings suggest that CT and MRI have comparable performance for detecting small PDACs and should be considered complementary rather than competing imaging modalities.

Magnetic resonance cholangiopancreatography (MRCP) provides excellent visualization of the pancreatic and biliary ducts without the need for contrast media. Simultaneous dilatation of the pancreatic duct and common bile duct, known as the “double-duct sign”, is well-demonstrated by MRCP and is highly suggestive of PDAC [25].

Diffusion-weighted imaging (DWI), a functional MRI technique, has also been investigated as an adjunct to conventional MRI for the diagnosis of PDAC [32,33]. Previous studies have reported excellent diagnostic performance, with a sensitivity of 96.2% and a specificity of 98.6% [34]. However, the tumors evaluated in these studies ranged from 16 to 49 mm in diameter (mean, 28 mm) [34], indicating that most were already relatively large at the time of diagnosis.

Takakura et al. compared DWI and MDCT for the detection of pancreatic cancer in a high-risk population with main pancreatic duct dilatation and found comparable diagnostic performance between the two modalities [33]. They suggested that the combination of MRCP and DWI enables both the identification of high-risk individuals and tumor detection using a single, non-contrast imaging examination. Nevertheless, although the mean tumor size was 31.6 ± 12.5 mm (range, 15–77 mm), several small, potentially curable tumors measuring less than 2 cm were not detected by DWI [33].

Wan et al. also evaluated the utility of DWI for PDAC with different histological grades of differentiation [35]. However, tumors smaller than 1 cm were excluded because a reliable evaluation of such small lesions was considered technically difficult.

Taken together, although DWI has shown promising diagnostic performance for PDAC, most published studies have focused on relatively advanced tumors rather than early-stage or small lesions [32,34,35]. Accordingly, Robertis et al. reviewed the available evidence and concluded that additional studies are required to determine the clinical value of DWI for the detection of small PDACs [32].

Compared with CT, MRI has the advantage of avoiding ionizing radiation and eliminating the risk of allergic reactions to iodinated contrast agents required for dynamic CT examinations. Therefore, assuming comparable diagnostic accuracy, MRI may theoretically be a more suitable modality for pancreatic cancer screening in high-risk individuals [33]. However, its widespread use is limited by higher cost, lower availability, the need for specialized expertise, and lower spatial resolution than CT [26].

### 2.5. Endoscopic Ultrasonography

Tumor size is one of the most important prognostic factors in pancreatic ductal adenocarcinoma (PDAC). Egawa et al. reported that patients with invasive PDAC measuring 10 mm or less (TS1a) had a significantly higher 5-year survival rate (80%) than those with tumors larger than 10 mm (TS1b or greater; 50%) [36]. Subsequently, Kanno et al. reported estimated 10-year overall survival rates after surgical resection of 94.7%, 93.8%, and 78.9% for Stage 0, Stage I (TS1a), and Stage I (TS1b), respectively [17]. These findings emphasize that detecting PDAC at a tumor size of ≤10 mm is critical for improving patient outcomes.

Yoshida et al. reviewed previous studies evaluating endoscopic ultrasonography (EUS) for the detection of small pancreatic lesions and concluded that EUS is more sensitive than other imaging modalities for detecting small PDACs [37]. For pancreatic lesions smaller than 30 mm, the reported sensitivities of EUS, CT, and magnetic resonance imaging (MRI) are 93%, 53%, and 67%, respectively [38]. Similarly, for tumors measuring 20 mm or less, EUS demonstrated significantly higher sensitivity than contrast-enhanced CT (94.4% vs. 50.0%) [39]. Furthermore, for pancreatic cancers measuring 10 mm or less, EUS achieved a sensitivity exceeding 80%, whereas the reported sensitivities of ultrasonography (US), CT, and positron emission tomography (PET) ranged from 17% to 70%, 33% to 75%, and approximately 50%, respectively [40].

Canto et al. evaluated screening in individuals at high risk for pancreatic cancer and reported that CT, MRI, and EUS detected pancreatic abnormalities in 11.0%, 33.3%, and 42.6% of participants, respectively [41]. They concluded that the surveillance of asymptomatic high-risk individuals frequently identifies small pancreatic cysts and curable, non-invasive high-grade precursor lesions, and that EUS and MRI are more sensitive than CT for detecting pancreatic abnormalities. Likewise, Lami et al. reviewed the literature and reported that EUS is capable of detecting focal pancreatic lesions as small as 2–5 mm [42].

Although EUS provides the highest sensitivity for detecting small pancreatic lesions, it has limitations for disease staging. Costache et al. reported that EUS is the most accurate modality for locoregional staging of pancreatic cancer but cannot reliably detect distant metastases, particularly extra-abdominal disease [19]. Therefore, cross-sectional imaging with CT or MRI remains essential for comprehensive staging.

Hanrinck et al. compared EUS and MRI for pancreatic cancer surveillance in high-risk individuals and concluded that the two modalities are complementary rather than interchangeable [43]. EUS showed superior sensitivity for detecting small solid lesions, whereas MRI was more sensitive for identifying cystic lesions regardless of size. These findings support the combined use of EUS and MRI in surveillance programs for individuals at increased risk of pancreatic cancer.

An additional advantage of EUS is its excellent negative predictive value (NPV) [44]. Two prospective studies with follow-up periods of 23.9 and 25 months, respectively, reported that none of the patients with negative EUS findings subsequently developed pancreatic cancer, corresponding to an NPV of 100% [45,46]. Furthermore, EUS enables tissue acquisition through endoscopic ultrasound-guided fine-needle aspiration (EUS-FNA) or fine-needle biopsy (EUS-FNB), allowing for histopathological confirmation of pancreatic lesions [46]. This capability has substantially improved the accuracy of diagnosing pancreatic cancer and facilitates earlier therapeutic intervention.

Technical aspects of EUS-guided tissue acquisition have been addressed in the guidelines of the European Society of Gastrointestinal Endoscopy (ESGE) [47]. For sampling solid pancreatic masses, both 22-gauge and 25-gauge needles are recommended. In addition, the ESGE recommends that pathological evaluation should include histological preparations, such as cell blocks or formalin-fixed paraffin-embedded tissue specimens, rather than relying solely on smear cytology (low-quality evidence, weak recommendation).

Despite the excellent diagnostic performance of EUS, the detection of small invasive PDAC does not necessarily guarantee a favorable prognosis. Even PDACs measuring less than 10 mm (TS1a) may already be classified as Stages II–IV at diagnosis [36], and the reported 5-year survival rate for TS1a tumors remains approximately 80.5% [36]. Once PDAC has invaded beyond the ductal epithelium into the pancreatic parenchyma, metastatic potential already exists, even when the tumor is very small.

Therefore, to achieve a complete cure, pancreatic cancer should ideally be detected before invasive carcinoma develops—that is, at Stage 0, corresponding to high-grade pancreatic intraepithelial neoplasia/carcinoma in situ (HG-PanIN/CIS). However, HG-PanIN/CIS itself cannot be directly visualized by any currently available imaging modality. Instead, diagnosis relies on the identification of secondary imaging findings that suggest the presence of these non-invasive precursor lesions.

Although high-grade pancreatic intraepithelial neoplasia (HG-PanIN) and carcinoma in situ (CIS) are not strictly synonymous in all pathological classification systems, these terms are used interchangeably throughout this review because of their closely related biological behavior and clinical significance.

## 3. Newly Developed Diagnostic Method

### 3.1. Focal Pancreatic Parenchymal Atrophy

In 2006, Brune et al. reported that some patients with a strong family history of pancreatic cancer developed numerous noninvasive epithelial precursor lesions, including pancreatic intraepithelial neoplasia (PanIN) and intraductal papillary mucinous neoplasm (IPMN), which were associated with lobular atrophy of the pancreatic parenchyma [48]. They described a spectrum of parenchymal atrophy associated with PanIN lesions and noted that these atrophic changes predominantly involved individual lobular units, with each affected lobule showing relatively uniform histological changes. A severely atrophic lobule could be located immediately adjacent to a histologically normal lobule. In the most advanced cases, marked acinar cell loss resulted in the near-complete disappearance of acinar tissue. These lobules consisted of a centrally located, mildly dilated duct surrounded by aggregates of islets of Langerhans embedded within fibro-fatty connective tissue. Furthermore, these pathological changes corresponded to chronic pancreatitis-like findings on endoscopic ultrasonography (EUS), including ductal abnormalities (ectasia, irregularity, and sacculation) and parenchymal abnormalities (heterogeneity and lobularity) [48]. These lesions were reported to harbor *KRAS* mutations [48].

In 2017, Kikuyama et al. reported three cases of high-grade PanIN/carcinoma in situ (HG-PanIN/CIS) accompanied by partial pancreatic parenchymal atrophy adjacent to the malignant lesion, which was detectable on computed tomography (CT) and magnetic resonance imaging (MRI) [49]. Histopathological examination demonstrated that the acinar lobules surrounding or adjacent to ducts containing HG-PanIN/CIS were atrophic and replaced by adipose tissue.

In 2020, Nakahodo et al. introduced the term focal pancreatic parenchymal atrophy (FPPA) to describe this characteristic imaging finding and demonstrated its association with HG-PanIN/CIS [50]. According to their definition, FPPA is identified on CT or MRI as a focal, asymmetrical, and irregular defect of the pancreatic parenchyma relative to the main pancreatic duct, typically accompanied by an irregular pancreatic surface [50]. Although Brune et al. did not describe the radiological findings of lobular atrophy on CT or MRI, FPPA is considered to represent the imaging correlate of lobular atrophy and may serve as an important clue for the detection of HG-PanIN/CIS.

**Figure 1:**
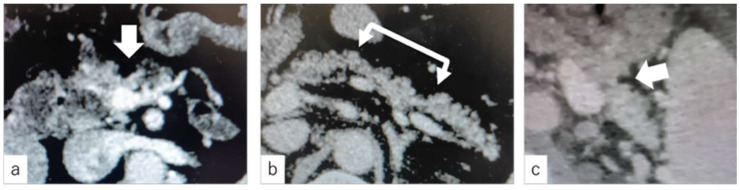
Types of focal pancreatic parenchymal atrophy (FPPA). (**a**) A Scheme showing types of FPPA: cave-in ((**a**) arrow), slimness ((**b**) square arrow), and slit ((**c**) arrow).

**Figure 2:**
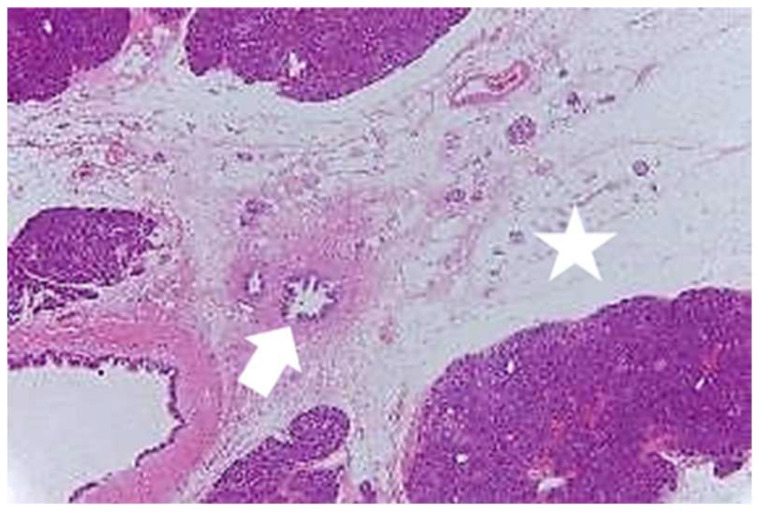
Histopathological findings of high-grade PanIN/carcinoma (arrow) in situ with focal pancreatic parenchymal atrophy with fat replacement and fibrosis (star). (H.E. ×40).

FPPA exhibits three distinct morphological patterns: cave-in, characterized by focal indentation of the pancreatic surface; slimness, characterized by longitudinal but localized atrophy of the pancreatic parenchyma with a shaggy appearance; and slit, characterized by a wedge-shaped defect of the pancreatic parenchyma (Figure 1). Among these, the slimness type was the most frequently observed. Histopathological examination of FPPA demonstrated predominant fibrosis, predominant fatty replacement, or a combination of both in the pancreatic parenchyma surrounding or adjacent to HG-PanIN/CIS (Figure 2).

Moreover, Nakahodo et al. investigated the prevalence of focal pancreatic parenchymal atrophy (FPPA) in patients with histologically confirmed HG-PanIN/CIS and reported that FPPA was detected in 55.6% of the patients, demonstrating a higher sensitivity for detecting HG-PanIN/CIS than main pancreatic duct stricture (MPS), which had a sensitivity of 44.0% [50]. They concluded that FPPA is an important secondary imaging finding for the diagnosis of early-stage pancreatic ductal adenocarcinoma (PDAC), particularly HG-PanIN/CIS [50].

Yamao et al. evaluated pancreatic parenchymal atrophy using multidetector computed tomography (MDCT) [51]. They found that the incidence of FPPA, defined as partial pancreatic parenchymal atrophy corresponding to the distribution of MPS, was significantly higher in patients with small PDAC than in those with benign MPS (45.8% vs. 7.1%, *p* < 0.01). They concluded that FPPA may serve as an imaging marker of pancreatic malignancy, particularly HG-PanIN.

Kikuyama et al. reported that among patients with FPPA, serial pancreatic juice aspiration cytologic examination (SPACE) yielded positive cytological findings in 46% [52]. Furthermore, 65% of the patients with positive SPACE findings underwent surgical resection including the entire FPPA lesion, and histopathological examination confirmed HG-PanIN/CIS in the resected specimens. In addition, they investigated the relationship between the size of FPPA and the histological grade of PanIN and demonstrated that an FPPA area > 270 mm^2^ was significantly associated with HG-PanIN [53]. These findings suggest that patients with FPPA, particularly those with large lesions, are appropriate candidates for SPACE.

Miki et al. investigated not only the diagnosis of carcinoma in situ (CIS), but also its intraductal extension in patients with FPPA and upstream pancreatic atrophy (UPA) secondary to MPS [54]. They demonstrated that the coexistence of FPPA and UPA was associated with the lateral extension of early-stage pancreatic cancer along the main pancreatic duct.

A nationwide multicenter study of early pancreatic cancer in Japan summarized the clinical characteristics of patients with PDAC, including established risk factors such as diabetes mellitus, tobacco use, IPMN, and heavy alcohol consumption [17]. The study also described imaging findings useful for diagnosing early-stage PDAC, including main pancreatic duct dilatation, MPS, pancreatic masses, and focal fatty changes of the pancreatic parenchyma. The focal fatty changes described in this study correspond to FPPA. The prevalence of this finding was 42.0% in Stage I PDAC and 41.8% in Stage 0 PDAC. Consistent with the findings of Nakahodo et al. and Kanno et al., FPPA has been recognized as an important imaging sign suggestive of HG-PanIN/CIS.

However, pancreatic atrophy may also occur as part of the aging process. Chronic pancreatitis frequently causes pancreatic atrophy, and diffuse fatty replacement of the pancreas is commonly observed in patients with diabetes mellitus or disorders of lipid metabolism. Therefore, FPPA associated with HG-PanIN/CIS should be distinguished from these benign conditions. Prospective studies are needed to establish reliable diagnostic criteria for this differentiation.

Conventionally, MPS has been regarded as an important imaging finding suggestive of malignancy in high-risk patients without an obvious pancreatic mass. Accordingly, the nationwide multicenter study in Japan reported that 83.0% of patients with Stage 0 PDAC had MPS [17]. However, Kalady et al. evaluated patients with MPS whose final diagnoses were established by cytology, histology, or clinical follow-up and classified them as having benign or malignant disease [55]. Among the 356 patients, 218 (61%) had isolated MPS without an accompanying common bile duct (CBD) stricture. Only 12% of patients with isolated MPS had malignant disease, whereas 79% of patients with MPS accompanied by CBD stricture were diagnosed with malignancy. These findings indicate that although MPS is an important indicator of pancreatic malignancy, isolated MPS alone has relatively limited specificity.

Several studies have further investigated imaging features that distinguish malignant from benign MPS. Miura et al. demonstrated that focal pancreatic atrophy with fatty replacement was significantly associated with malignancy in patients with MPS [56]. Similar findings were reported by Satoh et al. [57]. In contrast, Mahdi et al. noted that the large-duct type of chronic pancreatitis may also be associated with pancreatic atrophy [58], although their study did not distinguish between diffuse and focal atrophy. Collectively, these findings suggest that FPPA, rather than pancreatic atrophy in general, is the imaging abnormality most strongly associated with malignant pancreatic lesions.

**Figure 3:**
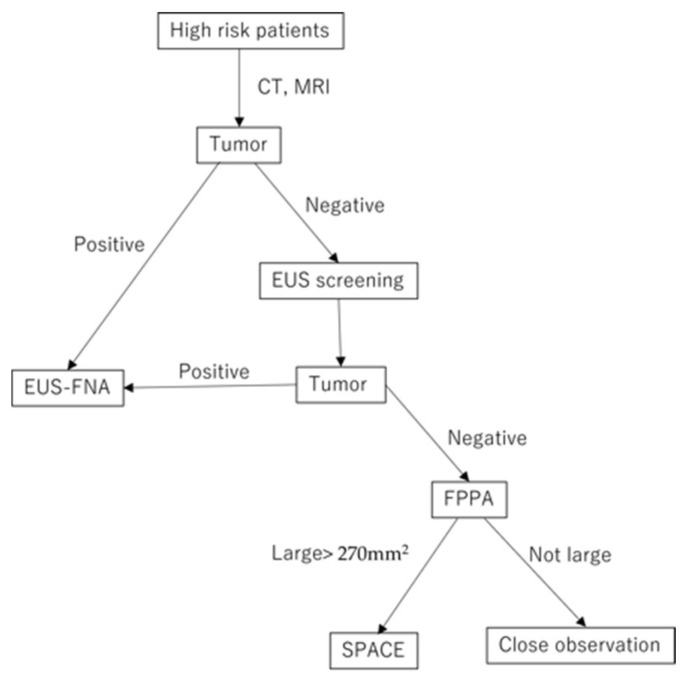
Proposal of an algorithm for diagnosing high-grade pancreatic intraepithelial neoplasia/carcinoma in situ. CT, computer tomography; MRI, magnetic resonance imaging; EUS, endoscopic ultrasonography; EUS-FNA, EUS-guided fine needle aspiration; FPPA, focal pancreatic parenchymal atrophy; SPACE, serial pancreatic juice aspiration cytologic examination.

FPPA is increasingly recognized as an important imaging marker of HG-PanIN/CIS, and an FPPA area > 270 mm^2^ appears to be particularly predictive of high-grade lesions [53]. Therefore, CT or MRI should be performed in individuals at high risk for PDAC, including those with a family history of PDAC, pancreatic cystic lesions, newly diagnosed or rapidly worsening diabetes mellitus, heavy smoking, or elevated serum carbohydrate antigen 19-9 (CA19-9). In patients without an identifiable pancreatic mass on endoscopic ultrasonography (EUS), the presence of a large FPPA (>270 mm^2^) should prompt the consideration of further diagnostic evaluation, including SPACE (Figure 3).

Serum CA19-9 also plays an important role in the detection of PDAC. A systematic review reported a median sensitivity of 79% and a median specificity of 82% for PDAC detection [59]. Another study reported a sensitivity of 76.1% for diagnosing early-stage PDAC [60]. However, the interpretation of CA19-9 requires the careful consideration of confounding factors, including cholestasis and Lewis antigen-negative status. Nevertheless, markedly elevated CA19-9 levels, particularly those exceeding 74 U/mL, should prompt further diagnostic evaluation for PDAC [61].

### 3.2. Acinar-Ductal Metaplasia

Some evidence from both human studies and animal models supports the hypothesis that PanIN develops first, leading to the obstruction of small pancreatic ducts, which subsequently results in lobulocentric atrophy and acinar-to-ductal metaplasia (ADM) [62]. ADM is a reversible process that enables the regeneration of pancreatic acinar tissue in response to chronic inflammation or cellular injury. However, in the presence of sustained oncogenic *KRAS* activation, ADM may become persistent, and reprogrammed acinar cells can give rise to PanIN lesions that subsequently progress to pancreatic ductal adenocarcinoma (PDAC) [63].

ADM is frequently accompanied by atrophy of the acinar lobules surrounding ducts containing PanIN lesions [63,64,65]. Obstruction of small pancreatic ducts by PanIN has been proposed as one of the mechanisms responsible for this lobular atrophy [48,62]. However, the precise mechanism underlying the development of pancreatic parenchymal atrophy has not yet been fully elucidated.

The lobular atrophy associated with precursor lesions provides a pathological explanation for the chronic pancreatitis-like changes observed in the pancreas. Furthermore, the patchy distribution of these atrophic changes suggests a potential imaging target for the detection of early pancreatic neoplasia in individuals at high risk for PDAC [48].

Kibe et al. defined acinar atrophy within the invasive front of PDAC as cancer-associated acinar atrophy (CAAA) [64]. They further demonstrated that ADM-like lesions accompanied by acinar atrophy and desmoplastic stromal reactions at the invasive front contribute to the formation of a tumor microenvironment that promotes the local invasion of pancreatic cancer [64].

### 3.3. Serial Pancreatic-Juice Aspiration Cytologic Examination

Pancreatic juice cytology has long been used as a conventional method for diagnosing pancreatic ductal adenocarcinoma (PDAC). Although it has high specificity, the sensitivity of conventional pancreatic juice cytology performed during endoscopic retrograde cholangiopancreatography (ERCP) is relatively low, at 36.4% [66]. Based on the concept that repeated cytological examination of pancreatic juice could improve the diagnostic yield, serial pancreatic juice aspiration cytologic examination (SPACE) using an endoscopic nasopancreatic drainage (ENPD) catheter was developed [67]. In this technique, an ENPD catheter is placed to enable the repeated collection of pancreatic juice for serial cytological examination.

Iiboshi et al. collected at least 1 mL of pancreatic juice five or six times at 2-h intervals by vacuum aspiration through an ENPD catheter left in place until the following morning [67]. Using this protocol, SPACE achieved a sensitivity of 100%, a specificity of 83.3%, and an overall diagnostic accuracy of 95% for PDAC, including carcinoma in situ.

Similarly, Mikata et al. evaluated SPACE by placing an ENPD catheter for up to three days and performing six serial cytological examinations [68]. The sensitivity, specificity, positive predictive value, negative predictive value, and overall diagnostic accuracy for pancreatic cancer were 80%, 100%, 100%, 71%, and 87%, respectively. The sensitivity was significantly higher than that of conventional pancreatic juice cytology (*p* = 0.0001) [68].

Sagami et al. performed SPACE by collecting pancreatic juice that accumulated in the ENPD catheter every 3 h, for a total of six collections, from 18:00 until 09:00 the following morning [66]. The sensitivity, specificity, and overall diagnostic accuracy were 77.3%, 93.5%, and 86.7%, respectively.

Collectively, these studies demonstrate that SPACE provides substantially greater diagnostic sensitivity than conventional pancreatic juice cytology while maintaining high specificity, making it one of the most reliable methods for diagnosing Stage 0 PDAC, including HG-PanIN/CIS [69,70].

However, SPACE requires the placement of an ENPD catheter, necessitating hospitalization for several days and carrying a risk of post-ERCP pancreatitis (PEP). Although the overall incidence of ERCP-related adverse events is relatively low and most cases of PEP are mild [53], ERCP remains an invasive procedure that should be performed only in carefully selected patients.

To reduce the risk of PEP, Mouri et al. investigated whether the use of a smaller-diameter ENPD catheter could decrease procedure-related complications [71]. They found that the incidence of PEP was significantly lower in patients who received a 4-Fr catheter than in those who received a 5-Fr catheter (4.1% vs. 12.4%, respectively; *p* = 0.021). Multivariate analysis demonstrated that use of a 5-Fr catheter was associated with a 3.7-fold higher risk of PEP than the use of a 4-Fr catheter (*p* = 0.019). They concluded that a 4-Fr ENPD catheter provides diagnostic performance comparable to that of a 5-Fr catheter while significantly reducing the risk of PEP [71]. Reducing the incidence of PEP would improve the safety and clinical applicability of SPACE.

In addition to catheter size, several non-endoscopic preventive strategies may further reduce the risk of PEP. These include the prophylactic administration of rectal nonsteroidal anti-inflammatory drugs (NSAIDs) and aggressive peri-procedural intravenous hydration, both of which have been shown to decrease the incidence of PEP after ERCP [72]. Further prospective studies are warranted to evaluate whether combining these preventive measures with SPACE can improve its safety and facilitate its wider clinical application.

### 3.4. Association Between FPPA and PDAC

**Figure 4:**
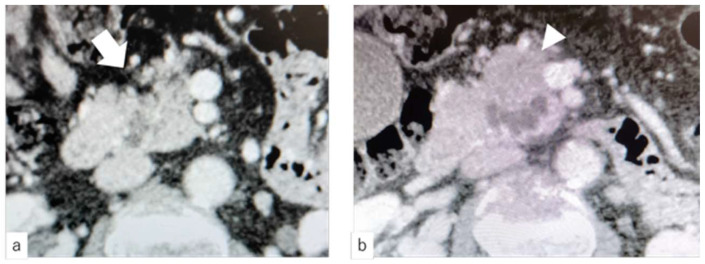
Pancreatic ductal adenocarcinoma development ((**b**) arrow head) adjacent to the pre-exiting focal pancreatic parenchymal atrophy ((**a**) arrow).

FPPA is considered to reflect the presence of PanIN, which progresses to pancreatic ductal adenocarcinoma (PDAC) through high-grade PanIN (HG-PanIN) [73]. In many patients with PDAC, the tumor develops adjacent to a pre-existing FPPA, with a mean distance of 7.93 mm between the estimated tumor center and the margin of the FPPA [74]. As the tumor enlarges, it appears to replace the area of parenchymal loss represented by the FPPA. These observations suggest that FPPA may represent either a precursor lesion or one of the earliest imaging manifestations of pancreatic carcinogenesis (Figure 4).

Nakahodo et al. investigated the prevalence of FPPA in patients who subsequently developed PDAC, defining FPPA as a focal, well-demarcated defect of the pancreatic parenchyma with an area greater than 50 mm^2^ [73]. Using this definition, they reported that FPPA was detectable before the diagnosis of PDAC in 27% of patients. Notably, all identified lesions were located in the pancreatic body or tail.

Similarly, Gonada et al. reported that focal parenchymal atrophy FPPA represents the earliest detectable imaging finding preceding PDAC and that the atrophic area was subsequently replaced by tumor at the time of diagnosis [75]. They also found that FPPA was significantly less frequent in patients with pancreatic head PDAC than in those with body/tail PDAC (10.4% vs. 43.6%, *p* < 0.001).

However, because PDAC most commonly arises in the pancreatic head, these findings appear inconsistent with the known anatomical distribution of the disease. Kikuyama et al. pointed out that this discrepancy is likely attributable to limitations of the imaging definition of FPPA rather than to the true distribution of precursor lesions [74]. The pancreatic head has unique anatomical characteristics that may lead to the under-recognition of FPPA. First, because it is a rounded three-dimensional structure, FPPA may extend along its curved surface, making its extent difficult to accurately assess on two-dimensional CT or MRI images. Second, the pancreatic head consists of embryologically distinct ventral and dorsal primordia. FPPA confined to either component or located at their junction may not produce an obvious surface contour defect and may therefore be overlooked on cross-sectional imaging.

To address these limitations, Kikuyama et al. revised the definition of FPPA and evaluated its prevalence and anatomical distribution before the development of PDAC [74]. In the pancreatic head, FPPA was defined as progressive focal pancreatic parenchymal atrophy regardless of lesion size, whereas in the pancreatic body and tail, the previously established morphological classification (slimness, cave-in, and slit) was applied. Using these criteria, FPPA was identified before the diagnosis of PDAC in approximately 83% of patients, with the pancreatic head representing the most frequent site (60%). These findings strongly support the concept that FPPA is an early imaging manifestation of pancreatic carcinogenesis and frequently precedes the development of PDAC, particularly in the pancreatic head.

Nevertheless, the use of different diagnostic criteria according to the anatomical location of the pancreas may cause confusion in clinical practice. Although accumulating evidence indicates that FPPA is a precursor imaging finding of PDAC [74], establishment of a simple and universally accepted definition is necessary to facilitate its widespread clinical application.

Similarly, Toshima et al. described several focal pancreatic abnormalities that preceded the development of PDAC, including focal pancreatic parenchymal atrophy, faint parenchymal enhancement, and abnormalities of the main pancreatic duct [76]. Among these findings, focal pancreatic atrophy was the most common, occurring in 37.9% of patients. In their study, pancreatic atrophy was defined as focal narrowing of the pancreatic parenchyma relative to the adjacent upstream and downstream pancreas. However, the anatomical distribution of the atrophic lesions was not reported.

Based on these studies, FPPA can be detected approximately 33–54 months before the diagnosis of PDAC [73,74,76]. Histopathologically, FPPA is thought to represent the imaging correlate of acinar-to-ductal metaplasia (ADM), a process induced by oncogenic *KRAS* activation in acinar cells [63]. Under persistent *KRAS* signaling, ADM progresses to PanIN and subsequently to PDAC [63,64]. ADM is accompanied by lobular acinar atrophy with fatty replacement and fibrosis [63,64], pathological changes that are visualized as FPPA on CT and MRI [50,51,52,53]. Therefore, the recognition of FPPA may provide a valuable opportunity for the detection of PDAC at an early, potentially curable stage.

PDAC is the most common malignant neoplasm of the pancreas. Huang et al. established culture conditions for generating both pancreatic ductal and acinar organoids from human stem cells and demonstrated distinct pathways of pancreatic tumorigenesis [77]. PanIN is believed to originate from acinar cells through ADM driven by oncogenic *KRAS* mutations, whereas intraductal papillary mucinous neoplasm (IPMN) develops from ductal epithelial cells and is primarily associated with *GNAS* mutations. Unlike PanIN-associated carcinogenesis, IPMN generally does not involve the acinar compartment and is therefore not associated with lobular atrophy. Consequently, when a cystic lesion is detected in the pancreas, the presence of FPPA may help distinguish a retention cyst associated with PanIN from an IPMN.

## 4. Conclusions

EUS is superior to US, CT, and MRI for detecting small PDACs. However, a small tumor does not necessarily represent early-stage disease, because even TS1a PDACs (≤10 mm in diameter) may already have progressed to Stages II–IV disease. In contrast, Stage 0 PDAC, defined as HG-PanIN/CIS, represents the earliest stage of pancreatic carcinogenesis and is not associated with metastatic spread. Therefore, improving the prognosis of PDAC depends on detecting these precursor lesions before the development of invasive cancer. Recognition of FPPA on CT or MRI, followed by SPACE in appropriate patients, may provide a valuable strategy for the early diagnosis of PDAC and ultimately improve patient outcomes.

## Data Availability

No new data were created or analyzed in this study. Data sharing is not applicable to this article.

## References

[1] Klein A.P. (2021). Pancreatic cancer epidemiology: Understanding the role of lifestyle and inherited risk factors. Nat. Rev. Gastroenterol. Hepatol..

[2] Leiphrakpam P.D., Chowdhury S., Zhang M., Bajaj V., Dhir M., Are C. (2025). Trends in the Global Incidence of Pancreatic Cancer and a Brief Review of its Histologic and Molecular Subtypes. J. Gastrointes Cancer.

[3] Ilic M., Ilic I. (2016). Epidemiology of pancreatic cancer. World J. Gastroenterol..

[4] Ferlay J., Soerjomataram I., Ervik M., Dikshit R., Eser S., Mathers C., Rebelo M., Parkin D.M., Forman D., Bray F. (2013). GLOBOCAN 2012 V1.0, Cancer Incidence and Mortality Worldwide: IARC CancerBase No. 11.

[5] (2025). Cancer Survival in Hospital-Based Cancer Registries. Cancer Information Service.

[6] Bengtsson A., Andersson R., Ansari D. (2020). The actual 5-year survivors of pancreatic ductal adenocarcinoma based on real-world data. Sci. Rep..

[7] Bockhorn M., Uzunoglu F.G., Mustapha Adham M., Clem Imrie C., Milicevic M., Sandberg A.A., Asbun H.J., Bassi C., Büchler M., Charnley R.M. (2014). Borderline resectable pancreatic cancer: A consensus statement by the International Study Group of Pancreatic Surgery (ISGPS). Surgery.

[8] Apte M.V., Xu Z., Pothula S., Goldstein D., Pirola R.C., Wilson J.S. (2015). Pancreatic cancer: The microenvironment needs attention too!. Pancreatology.

[9] Pothula S.P., Xu Z., Goldstein D., Pirola R.C., Wilson J.S., Apte M.V. (2016). Key role of pancreatic stellate cells in pancreatic cancer. Cancer Lett..

[10] Ren B., Cui M., Yang G., Wang H., Feng M., You L., Zhao Y. (2018). Tumor microenvironment participates in metastasis of pancreatic cancer. Mol. Cancer.

[11] Zeng S., Pöttler M., Grützmann R., Pilarsky C., Yang H. (2019). Chemoresistance in pancreatic cancer. Int. J. Mol. Sci..

[12] Kikuyama M., Kamisawa T., Kuruma S., Chiba K., Kawaguchi S., Terada S., Satoh T. (2018). Early diagnosis to improve the poor prognosis of pancreatic cancer. Cancers.

[13] Ciaravino V., D’Onofrio M. (2019). Pancreatic ultrasound: Stage of art. J. Ultrasound Med..

[14] Yamaguchi A., Kato N., Sugata S., Hamada T., Furuya N., Mizumoto T., Tamar Y., Kusunoki R., Kuwai T., Kouo H. (2022). Effectiveness of Abdominal Ultrasonography for Improving the Prognosis of Pancreatic Cancer during Medical Checkup: A Single Center Retrospective Analysis. Diagnostics.

[15] Ashida R., Tanaka S., Ioka T., Katayama K. (2017). Pancreatic cancer screening by abdominal ultrasonography. Suizo.

[16] Sofuni A., Tsuchiya T., Itoi T. (2020). Ultrasound diagnosis of pancreatic solid tumors. J. Med. Ultrason..

[17] Kanno A., Masamune A., Hanada K., Maguchi H., Shimizu Y., Ueki T., Hasebe O., Ohtsuka T., Nakamura M., Takenaka M. (2018). Multicenter study of early pancreatic cancer in Japan. Pancreatology.

[18] Sonal, Swati Bansal S., Malik M. (2025). The effectiveness of contrast-enhanced computed tomography for identifying diseases throughout the entire abdomen. Int. J. Radol Res..

[19] Costache M.I., Costache C.A., Dumitrescu C.I., Tica A.A., Popescu M., Baluta E.A., Anghel A.C., Saftoiu A., Dumitrescu D. (2017). Which is the best imaging method in pancreatic adenocarcinoma diagnosis and staging—CT, MRI or EUS?. Curr. Health Sci. J..

[20] Bileiro C., Andrade L., Santiago I., Marques R.M., Matos C. (2024). Imaging of pancreatic ductal adenocarcinoma—An update for all stages of patient management. Eur. J. Radiol. Open.

[21] Yoon S.H., Lee J.M., Cho J.Y., Lee K., Kim J.E., Moon S.K., Kim S.J., Baek J.H., Kim S.H., Kim S.H. (2011). Small (≤20 mm) pancreatic adenocarcinomas: Analysis of enhancement patterns and secondary signs with multiphasic multidetector CT. Radiology.

[22] Kitano M., Yoshida T., Itonaga M., Tamura T., Hatamaru K., Yamashita Y. (2019). Impact of endoscopic ultrasonography on diagnosis of pancreatic cancer. J. Gastroenterol..

[23] Kurita Y., Utsunomiya D., Kubota K., Koyama S., Hasegawa S., Hosono K., Irie K., Suzuki Y., Maeda S., Kobayashi N. (2024). Diagnostic Value of Contrast-Enhanced Dual-Energy Computed Tomography in the Pancreatic Parenchymal and Delayed Phases for Pancreatic Cancer. Tomography.

[24] LeBlanc M., Kang J., Costa A.F. (2023). Can we rely on contrast-enhanced CT to identify pancreatic ductal adenocarcinoma? A population-based study in sensitivity and factors associated with false negatives. Eur. Radiol..

[25] Al-Hawary M. (2016). Role of Imaging in Diagnosing and Staging Pancreatic Cancer. J. Natl. Compr. Cancer Netw..

[26] Kim J.H., Park S.H., Yu E.S., Kim M.H., Kim J., Byun J.H., Lee S.S., Hwang H.J., Hwang J.Y., Lee S.S. (2010). Visually isoattenuating pancreatic adenocarcinoma at dynamic-enhanced CT: Frequency, clinical and pathologic characteristics, and diagnosis at imaging examinations. Radiology.

[27] Schick V., Franzius C., Beyna T., Oei M.L., Schnekenburger J., Weckesser M., Domschke W., Schober O., Heindel W., Pohle T. (2008). Diagnostic impact of 18F-FDG PET–CT evaluating solid pancreatic lesions versus endosonography, endoscopic retrograde cholangio-pancreatography with intraductal ultrasonography and abdominal ultrasound. Eur. J. Nucl. Med. Mol. Imaging.

[28] Okano K., Kakinoki K., Akamoto S., Hagiike M., Usuki H., Yamamoto Y., Nishiyama Y., Suzuki Y. (2011). 18F-fluorodeoxyglucose positron emission tomography in the diagnosis of small pancreatic cancer. World J. Gastroenterol..

[29] Rijkers A.P., Valkema R., Duivenvoorden H.J., van Eijck C.H.J. (2014). Usefulness of F-18-fluorodeoxyglucose positron emission tomography to confirm suspected pancreatic cancer: A meta-analysis. Eur. J. Surg. Oncol. (EJSO).

[30] Jha P., Bijan B. (2015). PET/CT for pancreatic malignancy: Potential and pitfalls. J. Nucl. Med. Technol..

[31] Pu Y., Wang C., Zhao S., Xie R., Zhao L., Li K., Yang C., Zhang R., Tian Y., Tan L. (2021). The clinical application of 18F-FDG PET/CT in pancreatic cancer: A narrative review. Transl. Cancer Res..

[32] Robertis R.D., Martini P.T., Demozzi E., Corso F.D., Bassi C., Pederzoli P., D’Onofrio M. (2015). Diffusion-weighted imaging of pancreatic cancer. World J. Radiol..

[33] Takakura K., Sumiyama K., Munakata M., Ashida H., Arihiro S., Kakutani H., Tajiri H. (2011). Clinical usefulness of diffusion-weighted MR imaging for detection of pancreatic cancer: Comparison with enhanced multidetector-row CT. Abdom. Imaging.

[34] Ichikawa T., Erturk S.M., Motosugi U., Sou H., Iino H., Araki T., Fujii H. (2007). High–b value diffusion-weighted MRI for detecting pancreatic adenocarcinoma: Preliminary results. Am. J. Roentgenol..

[35] Wang Y., Chen Z.E., Nikolaidis P., McCarthy R.J., Merrick L., Sternick L.A., Horowitz J.M., Yaghmai V., Miller F.H. (2011). Diffusion-weighted magnetic resonance imaging of pancreatic adenocarcinomas: Association with histopathology and tumor grade. J. Magn. Reson. Imaging.

[36] Egawa S., Toma H., Ohigashi H., Okusaka T., Nakao A., Hatori T., Maguchi H., Yanagisawa A., Tanaka M. (2012). Japan pancreatic cancer registry; 30 year anniversary: Japan Pancreas Society. Pancreas.

[37] Yoshida T., Yamashita Y., Kitano M. (2019). Endoscopic Ultrasound for Early Diagnosis of Pancreatic Cancer. Diagnostics.

[38] Müller M.F., Meyenberger C., Bertschinger P., Schaer R., Marincek B. (1994). Pancreatic tumors: Evaluation with endoscopic US, CT, and MR imaging. Radiology.

[39] Sakamoto H., Kitano M., Suetomi Y., Maekawa K., Takeyama Y., Kudo M. (2008). Utility of Contrast-Enhanced Endoscopic Ultrasonography for Diagnosis of Small Pancreatic Carcinomas. Ultrasound Med. Biol..

[40] Yamaguchi K., Okusaka T., Shimizu K., Furuse J., Ito Y., Hanada K., Shimosegawa T., Okazaki K. (2017). Committee for Revision of Clinical Guidelines for Pancreatic Cancer of the Japan Pancreas Society. Clinical practice guidelines for pancreatic cancer 2016 from the Japan pancreas society a synopsis. Pancreas.

[41] Canto M.I., Hruban R.H., Fishman E.K., Kamel I.R., Schulick R., Zhang Z., Topazian M., Takahashi N., Fletcher J., Petersen G. (2012). For the American Cancer of the Pancreas Screening (CAPS) Consortium. Frequent Detection of Pancreatic Lesions in Asymptomatic High-Risk Individuals: Screening for Early Pancreatic Neoplasia (CAPS 3 Study). Gastroenterology.

[42] Lami G., Biagini M.R., Galli A. (2014). Endoscopic ultrasonography for surveillance of individuals at high risk for pancreatic cancer. World J. Gastrointest. Endosc..

[43] Harinck F., Konings I.C.A.W., Kluijt I., Poley J.W., van Hooft J.E., van Dullemen H.M., Nio C.Y., Krak N.C., Hermans J.J., Aalfs C.M. (2016). A multicentre comparative prospective blinded analysis of EUS and MRI for screening of pancreatic cancer in high-risk individuals. Gut.

[44] Helmstaedter L., Riemann J.F. (2007). Endoscopic ultrasound and early diagnosis of pancreatic cancer. Am. J. Surg..

[45] Klapman J.B., Chang K.J., Lee J.G., Nguyen P. (2005). Negative predictive value of endoscopic ultrasound in a large series of patients with a clinical suspicion of pancreatic cancer. Am. J. Gastroenterol..

[46] Bhutani M.S., Koduru P., Joshi V., Saxena P., Suzuki R., Irisawa A., Yamao K. (2016). The role of endoscopic ultrasound in pancreatic cancer screening. Endosc. Ultrasound.

[47] Polkowski M., Jenssen C., Kaye P., Carrara S., Deprez P., Gines A., Esparrach G.F., Eisendrath P., Aithal G.P., Arcidiacono P. (2017). Technical aspects of endoscopic ultrasound (EUS)-guided sampling in gastroenterology: European Society of Gastrointestinal Endoscopy (ESGE) Technical Guideline March-2017. Endoscopy.

[48] Brune K., Abe T., Canto M., O’Malley L., Klein A.P., Maitra A., Adsay N.V., Fishman E.K., Cameron J.L., Yeo C.J. (2006). Multifocal neoplastic precursor lesions associated with lobular atrophy of the pancreas in patients having a strong family history of pancreatic cancer. Am. J. Surg. Pathol..

[49] Kikuyama M., Hanada K., Ueki T. (2015). Pancreatic carcinoma in situ presenting prominent fatty change of the pancreatic body on CT: Experiences from 3 cases. Suizo.

[50] Nakahodo J., Kikuyama M., Nojiri S., Chiba K., Yoshimoto K., Kamisawa T., Horiguchi S.I., Honda G. (2020). Focal parenchymal atrophy of pancreas: An important sign of underlying high-grade pancreatic intraepithelial neoplasia without invasive carcinoma, i.e., carcinoma in situ. Pancreatology.

[51] Yamao K., Takenaka M., Ishikawa R., Okamoto A., Yamazaki T., Nakai A., Omoto S., Kamata K., Minaga K., Matsumoto I. (2020). Partial pancreatic parenchymal atrophy is a new specific finding to diagnose small pancreatic cancer (≤10 mm) including carcinoma in situ: Comparison with localized benign main pancreatic duct stenosis patients. Diagnostics.

[52] Kikuyama M., Nakahodo J., Honda G., Horiguchi S., Suzuki M., Chiba K., Tabata H., Ome Y., Kamisawa T. (2021). Effectiveness of focal pancreatic parenchymal atrophy in diagnosing high-grade pancreatic intraepithelial neoplasia/carcinoma in situ. Med. Res. Arch..

[53] Kikuyama M., Nakahodo J., Honda G., Suzuki M., Horiguchi S.I., Chiba K., Tabata H., Ome Y., Uemura S., Kawamoto Y. (2023). Pancreatic duct epithelial malignancy suggested by large focal pancreatic parenchymal atrophy in cystic diseases of the pancreas. Pancreatology.

[54] Miki M., Masuda A., Takenaka M., Shiomi H., Iemoto T., Tsumura H., Tsujimae M., Toyama H., Sofue K., Ueshima E. (2024). SMT Study Group in Japan. Association of pancreatic atrophy patterns with intraductal extension of early pancreatic ductal adenocarcinoma: A multicenter retrospective study. J. Gastroenterol..

[55] Kalady M.F., Peterson B., Baillie J., Onaitis M.W., Abdul-Wahab O.I., Howden J.K., Jowell P.S., Branch S., Clary B.M., Pappas T.N. (2004). Pancreatic duct strictures: Identifying risk of malignancy. Ann. Surg. Oncol..

[56] Miura S., Kume K., Kikuta K., Hamada S., Takikawa T., Yoshida N., Hongo S., Tanaka Y., Matsumoto R., Sano T. (2020). Focal parenchymal atrophy and fat replacement are clues for early diagnosis of pancreatic cancer with abnormalities of the main pancreatic duct. Tohoku J. Exp. Med..

[57] Sato K., Shigekawa M., Yoshioka T., Yamamoto S., Matsumae T., Koizumi K., Sato Y., Okabe J., Kodama T., Hikita H. (2023). Main pancreatic duct stenosis without detecting tumor. Suizo.

[58] Mahdi M.B., Steinkohl E., Singh V.K., Drewes A.M., Frøkjær J.B., Olesen S.S. (2023). Clinical course of medically managed patients with large and small duct chronic pancreatitis. Clin. Transl. Gastroenterol..

[59] Goonetilleke K.S., Siriwardena A.K. (2007). Systematic review of carbohydrate antigen (CA 19-9) as a biochemical marker in the diagnosis of pancreatic cancer. Eur. J. Surg. Oncol..

[60] Luo G., Uo M., Jin K., Liu Z., Liu C., Cheng H., Lu Y., Jin K., Liu L., Long J. (2016). Optimize CA19-9 in detecting pancreatic cancer by Lewis and Secretor genotyping. Pancreatology.

[61] Nazli O., Bozdag A.D., Tansug T., Kir R., Kaymak E. (2000). The diagnostic importance of CEA and CA 19-9 for the early diagnosis of pancreatic carcinoma. Hepatogastroenterology.

[62] Shi C., Hong S.M., Lim P., Kamiyama H., Khan M., Anders R.A., Goggins M., Hruban R.H., Eshleman R.E. (2009). KRAS2 mutations in human pancreatic acinar-ductal metaplastic lesions are limited to those with PanIN: Implications for the human pancreatic cancer cell of origin. Mol. Cancer Res..

[63] Marstrand-Daucé L., Lorenzo D., Chassac A., Nicole P., Couvelard A., Haumaitre C. (2023). Acinar-to-Ductal Metaplasia (ADM): On the Road to Pancreatic Intraepithelial Neoplasia (PanIN) and Pancreatic Cancer. Int. J. Mol. Sci..

[64] Kibe S., Ohuchida K., Ando Y., Takesue S., Nakayama H., Abe T., Endo S., Koikawa K., Okumura T., Iwamoto C. (2019). Cancer-associated acinar-to-ductal metaplasia within the invasive front of pancreatic cancer contributes to local invasion. Cancer Lett..

[65] Parte S., Nimmakayala R.K., Batra S.K., Ponnusamy M.P. (2022). Acinar to ductal cell trans-differentiation: A prelude to dysplasia and pancreatic ductal adenocarcinoma. BBA-Rev. Cancer.

[66] Sagami R., Mizukami K., Nishikiori H., Sato T., Fujiwara S., Kawamoto Y., Ome Y., Honda G., Horiguchi S., Sato K. (2024). Pancreatic juice cytology for diagnosing invasive pancreatic carcinoma/high-grade pancreatic intraepithelial neoplasia without visible tumors on endoscopic ultrasound. Pancreatology.

[67] Iiboshi T., Hanada K., Fukuda T., Yonehara S., Sasaki T., Chayama K. (2012). Value of cytodiagnosis using endoscopic nasopancreatic drainage for early diagnosis of pancreatic cancer: Establishing a new method for the early detection of pancreatic carcinoma in situ. Pancreas.

[68] Mikata R., Ishihara T., Tada M., Tawada K., Saito M., Kurosawa J., Sugiyama H., Sakai Y., Tsuyuguchi T., Miyazaki M. (2013). Clinical usefulness of repeated pancreatic juice cytology via endoscopic naso-pancreatic drainage tube in patients with pancreatic cancer. J. Gastroenterol..

[69] Satoh T., Kikuyama M., Kawaguchi S., Kanemoto H., Muro H., Hanada K. (2017). Acute pancreatitis-onset carcinoma in situ of the pancreas with focal fat replacement diagnosed using serial pancreatic-juice aspiration cytologic examination (SPACE). Clin. J. Gastroenterol..

[70] Kuruma S., Kikuyama M., Chiba K., Yoshimoto K., Kamisawa T., Goro Honda G., Horiguchi S., Nakahodo J. (2020). Carcinoma in situ of the pancreas with pancreatic duct stricture persistent for 4 years diagnosed by serial pancreatic juice aspiration cytologic examination (SPACE). Clin. J. Gastroenterol..

[71] Mouri T., Sasaki T., Serikawa M., Ishigaki T., Ishii Y., Shimizu A., Tsuboi T., Kurihara K., Tatsukawa Y., Miyaki E. (2021). Clinical analysis of early-stage pancreatic cancer and proposal for a new diagnostic algorithm: A multicenter observational study. Diagnostics.

[72] Weiland C.J.S., Akshintala V.S., Singh A., Buxbaum J., Choi J.H., Elmunzer B.J., Fogel E.S., Lai J.H., Levenick J.M., Gardner T.B. (2024). Preventive measures and risk factors for post-ERCP pancreatitis: A systematic review and individual patient data meta-analysis. Dig. Dis. Sci..

[73] Nakahodo J., Kikuyama M., Fukumura Y., Horiguchi S., Chiba K., Tabata H., Suzuki M., Kamisawa T. (2022). Focal pancreatic parenchyma atrophy is a harbinger of pancreatic cancer and a clue to the intraductal spreading subtype. Pancreatology.

[74] Gonda M., Masuda A., Kobayashi T., Iemoto T., Kakuyama S., Ezaki T., Ikegawa T., Hirata Y., Tsumura H., Ogisu K. (2024). Temporal progression of pancreatic cancer computed tomography findings until diagnosis: A large-scale multicenter study. United Eur. Gastroenterol. J..

[75] Kikuyama M., Nakahodo J., Chiba K., Honda G. (2025). Focal pancreatic parenchymal atrophy could be a precursor of pancreatic cancer. Pancreatology.

[76] Toshima F., Watanabe R., Yoneda N., Yamamoto T., Sasahira N., Sasaki T., Matsuyama M., Minehiro K., Tateichi U., Gabata T. (2021). CT abnormalities of the pancreas associated with the subsequent diagnosis of clinical stage I pancreatic ductal adenocarcinoma more than 1 year later: A case-control study. Am. J. Roentgenol..

[77] Huang L., Desai R., Conrad D.N., Leite N.C., Akshinthala D., Lim C.M., Gonzalez R., Muthuswamy L.B., Gartner Z., Muthuswamy S.K. (2021). Commitment and oncogene-induced plasticity of human stem cell-derived pancreatic acinar and ductal organoids. Cell Stem Cell.

